# Evaluation of ocular involvement in patients with Hansen’s disease

**DOI:** 10.1371/journal.pntd.0008585

**Published:** 2020-09-21

**Authors:** Priscila Daiane Pavezzi, Rodrigo Bueno do Prado, Plinio Ângelo Boin Filho, Airton dos Santos Gon, Bruna Tuma, Marco Aurelio Fornazieri, Fabiana de Mari Scalone, Luciana Rigolin Mazoni Alves, Ricardo Hirayama Montero, Antonio Marcelo Barbante Casella

**Affiliations:** 1 Internal Medicine Department, Dermatology, Universidade Estadual de Londrina, Londrina, PR, Brazil; 2 Clinical Surgery Department, Ophthalmology, Universidade Estadual de Londrina, Londrina, PR, Brazil; 3 Clinical Surgery Department, Otorhinolaryngology, Universidade Estadual de Londrina, Londrina, PR, Brazil; JH-Institute of Molecular Medicine, INDIA

## Abstract

Hansen’s disease (HD) belongs to the group of neglected diseases and can cause physical deformities and disabilities, in addition to leading to social discrimination. Ocular involvement in HD is estimated at 70–75% worldwide. About 10–50% suffer from severe ocular symptoms and loss of vision occurs in approximately 5% of cases. Ocular changes may persist or worsen even after patients are considered cured and it is necessary to better understand these conditions in order to determine the need for additional public policies. The objective of this study was to identify the prevalence of ocular involvement in patients with HD at two specialist referral centers for treatment of the disease. A cross-sectional study was conducted with ophthalmological evaluations of patients with HD from June 2017 to June 2018. Diagnostic ocular findings, corrected visual acuity, and refractive error were described. Findings were correlated with patients’ clinical and epidemiological variables. A total of 86 patients were evaluated, with a mean age of 50.1 years, predominantly males (59.3%), and with multibacillary HD (92%). The prevalence of ophthalmologic changes was 100% and the most common were dysfunction of the Meibomian glands (89.5%) and dry eye syndrome (81.4%). Cataracts were observed in 22 patients (25.6%), but best corrected visual acuity was normal or near normal in 84 patients (97.7%) and there were no cases of bilateral blindness. Patients with some degree of physical disability had more ophthalmological alterations, involving both the ocular adnexa (p = 0.03) and the ocular globe (p = 0.04). Ocular involvement is common in patients with Hansen’s disease, reinforcing the importance of ophthalmologic examination in the evaluation and follow-up of these patients.

## Author summary

Leprosy–although use of this term is not recommended because of the negative stigma attached to it–also known as Hansen’s disease is one of the oldest diseases in the world. In modern times, we have identified the agent that causes it, we know how to diagnose it, and we know how to treat it. Nevertheless, thousands of people are still diagnosed late and even though they receive the treatment recommended, they suffer from sequelae that can be highly incapacitating. When discussing its clinical presentation, what springs to mind, even among the public, are the notorious, whitened, discolored patches of skin with sensory dysfunction, but ocular involvement caused by the *Mycobacterium leprae* bacteria is often ignored by patients and even health professionals. Few studies have been published worldwide reporting the true incidence of eye damage caused by Hansen’s disease and identifying which patients need special care for the eyes. It is extremely important to raise awareness and increase knowledge about these conditions, so that public policies on treatment and prevention can be established. This study found that 100% of the sample of patients with Hansen’s disease had some type of abnormality of the eyes or adnexa and that patients over the age of 50 and those with some degree of physical incapacity secondary to Hansen’s disease were at greatest risk. Another important finding was the high rate of Meibomian gland dysfunction (89.5%) and dry eye syndrome.

## Introduction

Leprosy, also known as Hansen’s disease (HD), is a chronic, infectious, contagious disease caused by *Mycobacterium leprae*. Contagion is caused by elimination of the bacillus via the airways of untreated patients, infecting susceptible individuals who come into close and prolonged contact [1–4]. It primarily involves the skin, peripheral nerves, and eyes [1, 2] and is the bacterial disease with the highest incidence of ocular involvement, compared with any other bacterial infection in humans [5–8]. At the end of 2018, the prevalence rate of HD was 0.24 per 10,000 population and 208,619 new cases were detected globally. The countries with greatest incidence are India, Brazil, and Indonesia, with 57.7%, 13.7%, and 8.2% respectively of all new cases worldwide in 2018. [9–12].

In 2018, Brazil registered 28,660 new cases of HD, 1,785 more than in the previous year. The most affected regions are the Northeast (40.9%), the Midwest (23.2%), the North (20.2%), the Southeast (12.9%) and the South (2.8%). Among the three southern states, Paraná has the largest number of new cases notified, with 550 patients, a number 12 times greater than the sum of all new cases reported in Europe in the same period. Londrina is the fourth most affected city in the state, with 18 new cases in 2018 [10].

In 1982, the World Health Organization (WHO) recommended multidrug therapy (MDT) with rifampicin, dapsone and clofazimine for treatment of HD and more than 16 million patients have been treated with MDT over the last 20 years [3]. Since these patients are recorded as treated, they are considered "cured" and are no longer counted as HD cases [2, 13]. This policy resulted in a rapid and massive reduction in the apparent prevalence of HD, but certain problems can even worsen after "cure" and this is the case of ocular involvement [14–16].

The most important ocular clinical manifestations are lagophthalmos, ectropion, entropion, trichiasis, corneal hypoesthesia, superficial punctate keratitis, corneal ulcers and/or opacity with reduced visual acuity, episcleritis, scleritis, scleral nodule, formation of “iris pearls” (pathognomonic), atrophy of the iris, iridocyclitis, chronic uveitis, and cataracts. The last of these is the principal cause of blindness among patients with HD, including those who have completed MDT. Secondary infections can also occur, as well as chorioretinopathy, due to the prolonged use of systemic corticosteroids for treatment of leprosy reactions [4, 7, 15–17].

The majority of vision loss secondary to HD is preventable or curable [18–21]. Despite this, patients with HD are rarely assessed by an ophthalmologist. The WHO system for classification of disabilities related to HD includes a category for grading involvement of the eyes among the three organs classified (eyes, hands, and feet) [22]. Training manuals are available, but, sadly, in the majority of programs the eyes are still being ignored or dealt with superficially [6, 16].

In 2016, the WHO launched its *Global Leprosy Strategy 2016–2020*: *Accelerating towards a leprosy-free world*, with the objective of reinvigorating efforts to control the disease and prevent disabilities. The strategy stresses the need to reduce visible deformities–also known as grade 2 impairments (G2I)–which include lagophthalmos, iridocyclitis, corneal opacity, and severe visual incapacity (vision: < 6/60; inability to count fingers at 6 meters) [23].

Patients with HD should have their eyes carefully examined, because the possibility of loss of vision combined with loss of tactile, thermal and painful sensation, leaves the patient extremely unprotected. The objectives of this study were therefore to evaluate the prevalence of ocular involvement among patients with HD and establish during which phase of the disease ocular involvement was most prevalent, whether during treatment or after treatment was completed, in addition to exploring clinical and epidemiological factors that could possibly be related to greater eye and adnexa involvement.

## Methods

### Study design and ethics

A cross-sectional study was conducted with data collection from June 2017 to June 2018. Approval was granted by the Human Research Ethics Committee at the Universidade Estadual de Londrina (decision number 2 089 988) and all patients gave informed consent in writing before enrollment on the study. For patients aged less than 18 years, parents’ consent was obligatory. Volunteers with HD ocular manifestations were advised about the necessary care with the eyes and treated.

### Sample, study location, inclusion criteria

The study participants were selected by convenience sampling, consecutively, at two specialist referral centers for treatment of HD in the city of Londrina, state of Paraná, south region of Brazil.

Londrina is the second largest city in the state of Paraná, with a population of 569,733 and a human development index of 0.778. The HD incidence rates in 2017 and 2018 was 6.27 and 3.16 per 100,000 inhabitants, respectively.

All patients diagnosed with HD, in the course of treatment or with treatment already completed, irrespective of whether they had ocular complaints, were invited to attend for an ophthalmological assessment at a private clinic. There was no exclusion criterion.

### Study variables

The descriptive variables, variables predictive of ocular involvement, and the patient data analyzed, can be didactically divided into epidemiological and clinical variables and ophthalmic examination variables.

The epidemiological and clinical variables are: sex, age, time since diagnosis, classification of disease spectrum, presence of relapse, bacilloscopy results at diagnosis, use of MDT (conventional or alternative), presence of leprosy reactions, presence of some degree of physical incapacity (DFI).

Regarding the disease spectrum, patients were classified into 3 groups according to the Madrid classification: tuberculoid (T), borderline (B) (or dimorphic), and virchowian (V) (or lepromatous). To facilitate the indication of treatment, the WHO recommends the operational classification in multibacillary (MB) and paucibacillary (PB), but it was decided to maintain the Madrid classification, as this was the one used in medical records of the patients in the study and takes into account the different pathophysiological and immunological aspects of the disease.

About the MDT, patients were enrolled who had completed or were undergoing treatment with traditional MDT (rifampicin, dapsone and clofazimine) or alternative MDT (ofloxacin, minocycline).

The DFI (according to the 1998 WHO score)^22^ considered was the highest degree presented by the patient during the follow-up by the attending physician, according to notes in the medical record, until the day of the eye examination.

The ophthalmic examination variables are: reported presence of ocular complaint, involvement of ocular adnexa (madarosis of eyelashes and eyebrows), eyelid involvement (lagophthalmos, trichiasis, and/or ectropion), obstruction of tear ducts, Meibomian gland dysfunction, dry eye syndrome (defined by a Schirmer`s test result < 5mm and/or presence of punctate keratitis), abnormal monofilament test result, pupil cycle time (PCT), intraocular pressure (IOP), eyeball abnormalities–subdivided into injuries to the surface of the eye (sclera, conjunctiva, cornea) and intraocular injuries (iris, vitreous chamber, retina). The term “ocular involvement” refers to the presence or alteration in any abnormality listed in the ophthalmic variables.

### Data collection

The sources consulted for epidemiological and clinical data collection were patients’ electronic medical records, available at the specialist treatment centers, and copies of the obligatory notifiable disease reporting forms completed at the time of diagnosis. Data from the ophthalmological assessment were taken from the examination protocol. Ophthalmological assessments were conducted for all patients by a single examiner (RBP, blinded to patients’ clinical data), on a single day, and were broken down into the following stages: to ask the patient about eye complaints, general examination and assessment of ocular motility, subjective refraction tests and determination of the best corrected visual acuity, biomicroscopy of the anterior segment, PCT measurement, Schirmer’s test, applanation tonometry, cycloplegia for mapping of the retina, and optical coherence tomography (OCT).

### Statistics

Quantitative data were expressed as means (standard deviation) and minimum and maximum values. Categorical variables were expressed as counts and percentages. Data on presence or absence of lesions of adnexa and eyeball were collapsed to compound outcomes and then dichotomized as mild involvement (zero or one finding of involvement) or intense (two or more findings of involvement). The statistical significance of observed differences was evaluated using the Fisher’s exact test. The strength of associations was expressed by odds ratios with respective 95% confidence intervals. Forest plots were used to illustrate the profile of distribution of events, arranging effects from mildest to most intense. Since this was an exploratory study, no attempt was made to adjust for multiplicity. Results with p< 0.05 were considered statistically significant. Data were analyzed using IBM-SPSS version 22.0 and graphs were plotted using SigmaPlot version 11.0.

## Results

### Patients characteristics

A total of 86 patients were recruited for the study. Fifty-one of the participants (59.3%) were male, the mean (±SD) age was 50.1±13.1 years (range: 17 to 81), and 51.2% were over the age of 50 years. The median (range) time since diagnosis of the disease was 2.1 (0 to 19) years, with 21 patients (24.4%) diagnosed 5 years or more previously, 65 (75.6%) within the preceding 5 years, and eight (9.6%) new cases. Forty (46.5%) patients had already had MDT withdrawn after being cured and were in follow-up, 46 (53.5%) were in treatment with MDT, 19 (22.1%) of whom were on alternative medication.

With regard to the classification of disease spectra at diagnosis, seven (8.1%) patients had tuberculoid HD, 47 (54.7%) had one of the borderline forms, and 32 (37.2%) had virchowian form. Bascilloscopy results were positive at the time of diagnosis in 53% of cases, and 23.3% of patients were relapsed. With regard to presence of leprosy reactions, 37 (43%) patients exhibited some type of reaction, with predominance of type 2 reaction, in 26 patients. The records showed that 57 (66.3%) patients had some type of DFI, but only five of these were due to ocular involvement.

### Ocular findings

Forty-nine (57%) patients reported some type of eye-related complaint. The most common were burning/stinging sensation, difficulty seeing, lacrimation, blurred vision, and photosensitivity. Evaluation of best corrected visual acuity revealed that 84 (97.7%) had normal or near-normal vision and just two (2.3%) patients exhibited subnormal vision, one of whom had vision loss in the contralateral eye. There were no cases of bilateral blindness. With relation to their refractional diagnoses, 43 patients (50%) had hyperopic astigmatism, 27 (31.4%) were emmetropic, seven (8.14%) were hyperopic, three (3.49%) were myopic, and six exhibited other forms of astigmatism.

Tables 1 and 2 lists the frequencies of ocular findings in the 86 patients studied. The most common findings were Meibomian gland dysfunction, found in 77 individuals (89.5%) and more frequent among patients over the age of 50 (p = 0.014); and dry eye syndrome, seen in 70 individuals (81.4%). Thirty-five (50%) of these patients had evaporative dry eye, 13 (19%) had low baseline tear production, and 22 patients (31%) had mixed forms.

**Table 1:** Ophthalmological abnormalities, by characteristics of patients with Hansen’s disease.

nº (%)	Ocular complaint		Madarosis		Eyelid abnormality^1^		Obstruction of lacrimal glands		MGD^2^	
	Yes	No	Yes	No	Yes	No	Yes	No	Yes	No
	n = 49	n = 37	n = 12	n = 74	n = 3	n = 83	n = 2	n = 84	n = 77	n = 9
Age > 50 years	24 (49.0)	20 (54.1)	10 (83.3)	34 (45.9)	3 (100)	41 (49.4)	1 (50.0)	43 (51.2)	43 (55.8)	1 (11.1)
Female	24 (49.0)	11 (29.7)	03 (25.0)	32 (43.2)	0 (0)	35 (42.2)	0 (0)	35(41.7)	32 (41.6)	3 (33.3)
Time since diagnosis										
< 1 year	13 (26.5)	15 (40.5)	3 (25.0)	25 (33.8)	0	28 (33.7)	0 (0.0)	28 (33.3)	24 (31.2)	4 (44.4)
1 to 5 years	23 (46.9)	14 (37.8)	7 (58.3)	30 (40.5)	2 (66.7)	35 (42.2)	1 (50.0)	36 (42.9)	32 (41.6)	5 (55.6)
≥ 5 years	13 (26.5)	8 (21.6)	2 (16.7)	19 (25.7)	1 (33.3)	20 (24.1)	1 (50.0)	20 (23.8)	21 (27.3)	0 (0.0)
Taking MDT	25 (51.0)	21 (56.8)	5 (41.7)	41 (55.4)	2 (66.7)	44 (53.0)	1 (50.0)	45 (53.6)	41 (53.2)	5 (55.6)
Disease classification										
T	5 (10.2)	2 (5.4)	2 (16.7)	5 (6.8)	0 (0.0)	7 (8.4)	0 (0.0)	7 (8.3)	6 (7.8)	1 (11.1)
B	26 (53.1)	21 (56.8)	5 (41.7)	42 (56.8)	2 (66.7)	54 (54.2)	0 (0.0)	47 (56.0)	41 (53.2)	6 (66.7)
V	18 (36.7)	14 (37.8)	5 (41.7)	27 (36.5)	1 (33.3)	31 (37.3)	2 (100)	30 (35.7)	30 (39.0)	2 (22.2)
Positive bacilloscopy result	20 (40.8)	13 (35.1)	2 (16.7)	31 (41.9)	0 (0.0)	33 (39.8)	0 (0.0)	33 (39.3)	30 (39.0)	3 (33.3)
Disease in relapse	11 (22.4)	9 (24.3)	4 (33.3)	16 (21.6)	1 (33.3)	19 (22.9)	1 (50.0)	19 (22.6)	19 (24.7)	1 (11.1)
Time on MDT										
6 months	2 (4.1)	1 (2.7)	0 (0.0)	3 (4.1)	0 (0.0)	3 (3.6)	0 (0.0)	3 (3.6)	2 (2.6)	1(11.1)
12 months	38 (77.6)	26 (70.3)	9 (75.0)	55 (74.3)	1 (33.3)	63 (75.9)	1 (50.0)	63 (75.0)	57 (74.0)	7 (77.8)
24 months	6 (12.2)	6 (16.2)	0 (0.0)	12 (16.2)	1 (33.3)	11 (13.3)	1 (50.0)	11 (13.1)	11 (14.3)	1 (11.1)
36 months	3 (6.1)	4 (10.8)	3 (25.0)	4 (5.4)	1 (33.3)	6 (7.2)	0 (0.0)	7 (8.3)	7 (9.1)	0 (0.0)
Taking alternative MDT	9 (18.4)	10 (27.0)	5 (41.7)	14 (18.9)	1 (33.3)	18 (21.7)	1 (50.0)	18 (21.4)	17 (22.1)	2 (22.2)
Has leprosy reactions	19 (38.8)	18 (48.6)	6 (50.0)	31 (41.9)	1 (33.3)	36 (43.4)	2 (100)	35 (41.7)	32 (41.6)	5 (55.6)
Disability present^3^	29 (59.2)	28 (75.7)	8 (66.70	49 (66.2)	1 (33.3)	56 (67.5)	2 (100)	55 (65.5)	53 (68.8)	4 (44.4)

T, Tuberculoid HD; B, Borderline HD; V, Virchowian HD; MDT, multidrug therapy.

^1^ trichiasis/ectropion/lagophthalmos

^2^ Meibomian gland dysfunction

^3^ Any grade of disability present.

**Table 2:** Ophthalmological abnormalities, by characteristics of patients with Hansen’s disease.

nº (%)	Abnormal monofilament test result		Dry eye syndrome		Eye surface injury^1^		Intraocular injury^2^	
	Yes	No	Yes	No	Yes	No	Yes	No
	n = 10	n = 76	n = 70	n = 16	n = 33	n = 53	n = 33	n = 53
Age > 50 years	9 (90.0)	35 (46.1)	39 (55.7)	5 (31.3)	21 (63.6)	23 (43.4)	22 (66.7)	22 (41.5)
Female	05 (50.0)	30 (39.5)	30 (42.9)	05 (31.3)	10 (30.3)	25 (47.2)	13 (39.4)	22 (41.5)
Time since diagnosis								
< 1 year	5 (50.0)	23 (30.3)	23 (32.9)	5 (31.3)	14 (42.4)	14 (26.4)	12 (36.4)	16 (30.2)
1 to 5 years	4 (40.0)	33 (43.4)	31 (44.3)	6 (37.5)	10 (30.3)	27 (50.9)	13 (39.4)	24 (45.3)
≥ 5 years	1 (10.0)	20 (26.3)	16 (22.9)	5 (31.3)	9 (27.3)	12 (22.6)	8 (24.2)	13 (24.5)
Taking MDT	4 (40.0)	42 (55.3)	38 (54.3)	8 (50.0)	18 (54.5)	28 (52.8)	22 (66.7)	24 (45.3)
Disease classification								
T	0 (0.0)	7 (9.2)	5 (7.1)	2 (12.5)	2 (6.1)	5 (9.4)	2 (6.1)	5 (9.4)
B	9 (90.0)	38 (50.0)	36 (51.4)	11 (68.8)	17 (51.5)	30 (56.6)	16 (48.5)	31 (58.5)
V	1 (10.0)	31 (40.8)	29 (41.4)	3 (18.8)	14 (42.4)	18 (34.0)	15 (54.5)	17 (32.1)
Positive bacilloscopy result	4 (40.0)	29 (38.2)	26 (37.1)	7 (43.8)	12 (36.4)	21 (39.6)	13 (39.4)	20 (37.7)
Disease in relapse	01 (10.0)	19 (25.0)	16 (22.9)	4 (25.0)	8 (24.2)	12 (22.6)	10 (30.3)	10 (18.9)
Time on MDT								
6 months	0 (0.0)	3 (3.9)	1 (1.4)	2 (12.5)	0 (0.0)	3 (5.7)	0 (0.0)	3 (5.7)
12 months	8 (80.0)	56 (73.7)	53 (75.7)	11 (68.8)	26 (78.8)	38 (71.7)	25 (75.8)	39 (73.6)
24 months	2 (20.0)	10 (13.2)	9 (12.9)	3 (18.8)	3 (9.1)	9 (17.0)	5 (15.2)	7 (13.2)
36 months	0 (0.0)	7 (9.2)	7 (10.0)	0 (0.0)	4 (12.1)	3 (5.7)	3 (9.1)	4 (7.5)
Taking alternative MDT	0 (0.0)	19 (25.0)	18 (25.7)	1 (6.3)	7 (21.2)	12 (22.6)	7 (21.2)	12 (22.6)
Has leprosy reactions	2 (20.0)	35 (46.10	32 (45.7)	5 (31.3)	12 (36.4)	25 (47.2)	14 (42.4)	23 (43.4)
Disability present^3^	6 (60.0)	51 (67.1)	49 (70.0)	8 (50.0)	24 (72.7)	33 (62.3)	25 (75.8)	32 (60.4)

T, Tuberculoid HD; B, Borderline HD; V, Virchowian HD; MDT, multidrug therapy.

^1^ abnormalities of the sclera/conjunctiva/cornea/pterygium

^2^ abnormalities of the iris/vitreous chamber/DVP/glaucoma/cataract

^3^ Any grade of disability present

**Fig 1:**
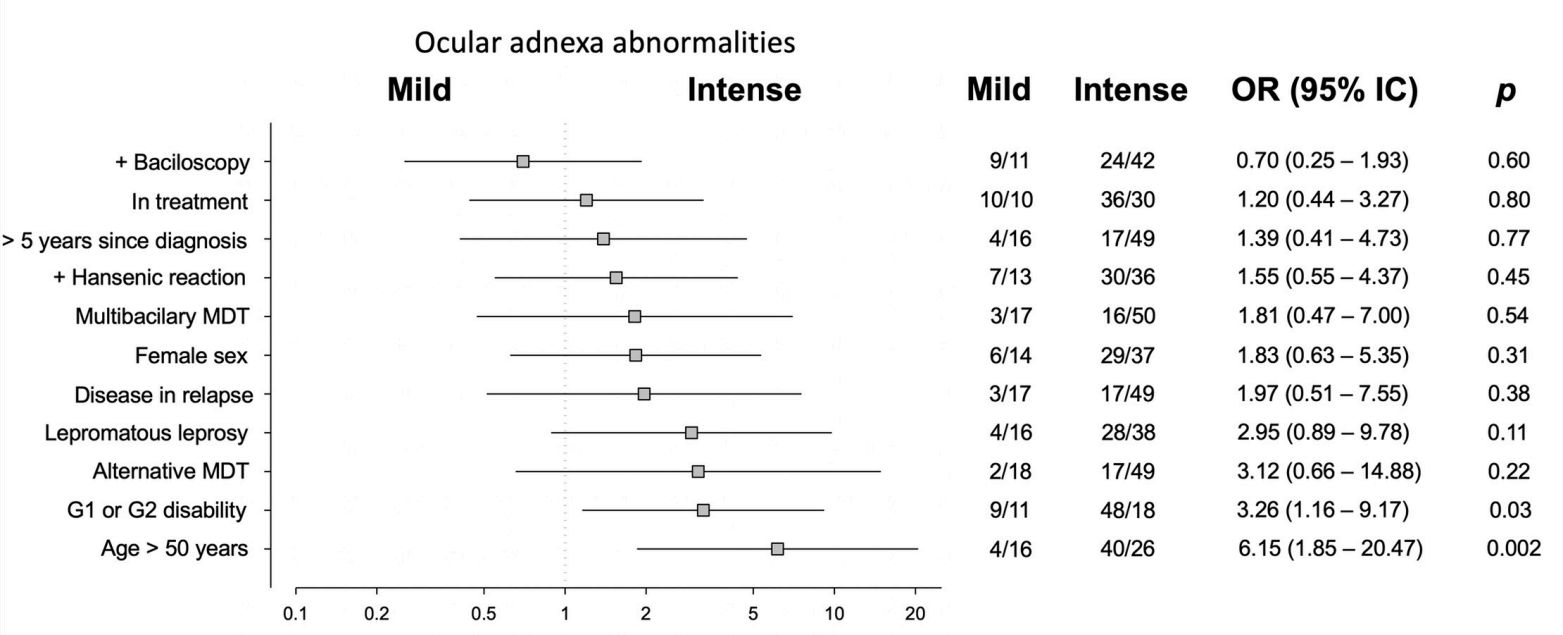
Univariate analysis illustrating associations between factors in patients with Hansen’s disease and presence of mild or intense abnormalities of the ocular adnexa. The values of odds ratios are shown in log scale with their respective 95% confidence intervals and with estimates of statistical significance (p) according to the Fisher’s exact test.

**Fig 2:**
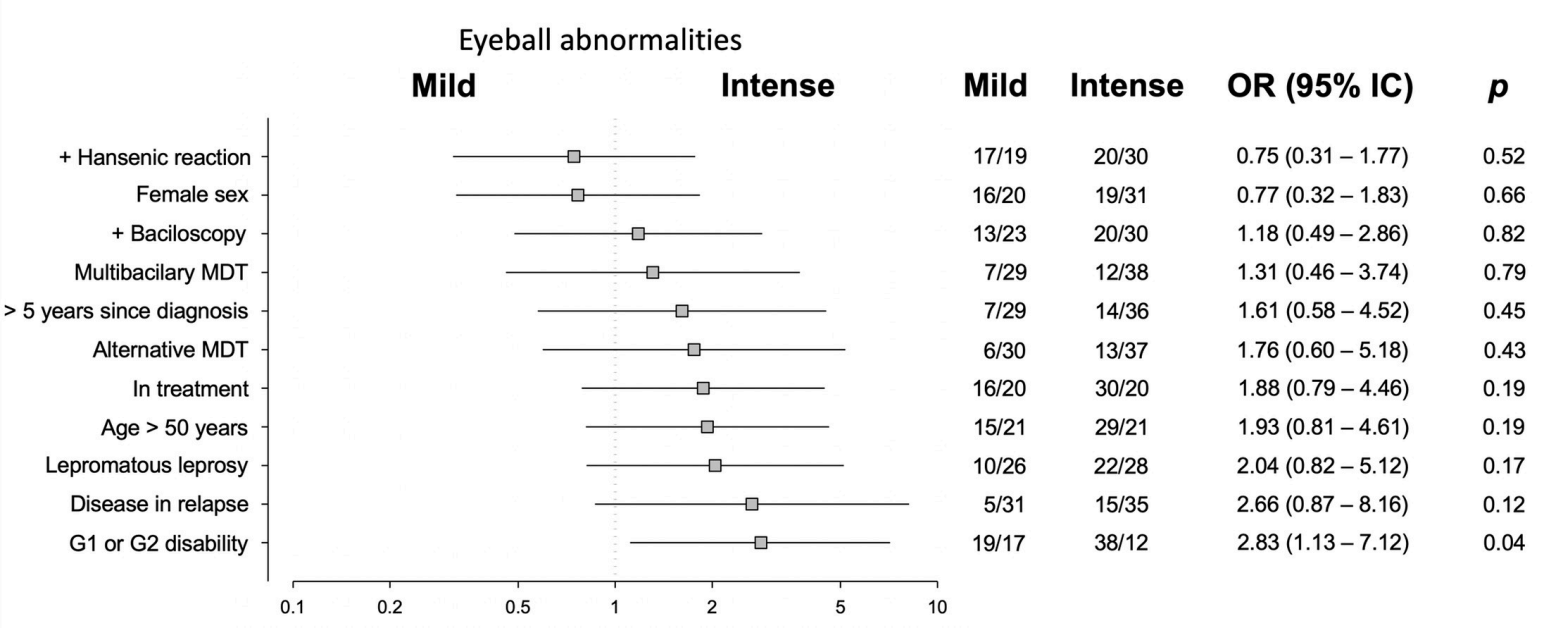
Univariate analysis illustrating associations between factors in patients with Hansen’s disease and presence of mild or intense abnormalities of the eyeball. The values of odds ratios are shown in log scale with their respective 95% confidence intervals and with estimates of statistical significance (p) according to the Fisher’s exact test.

The majority of patients had multiple ocular lesions, ranging from two patients with only one abnormality each to one patient with 14 abnormal findings in the ophthalmological examination. Fig 1 groups patients into two categories by ocular adnexa findings: mild, i.e., those with 2 abnormalities or fewer; and intense, including those who exhibited three or more adnexal abnormalities. An analogous system was used in Fig 2 for the eyeballs. When controlling for age (exact age in years) and sex, the odds ratios did not change significantly.

Among the 33 (38,4%) patients with eye surface injuries, 28 (32.5%) were conjunctival injuries; while among those with intraocular lesions, cataracts were found in 22 (25.6%) patients, eight (9.3%) had glaucoma, and five (5.8%) had atrophy of the iris. Retinal abnormalities were found in nine (10.5%) individuals.

When tested for PCT, patients exhibited times ranging from 0.685 seconds to 1.344 s, with a mean time of 0.903 s for the right eye and 0.892 s for the left eye. Mean IOP was 14 mmHg for both eyes, with a range of 6 to 40 mmHg.

Patients with greater time elapsed since diagnosis did not exhibit a greater occurrence of ocular abnormalities than those diagnosed more recently. There were also no differences between the prevalence rates of different ocular lesions according to sex, disease classification, presence of relapse, leprosy reactions, or use of alternative MDT. Patients with some type of DFI exhibited more ophthalmological abnormalities, with a three times greater chance of intense involvement, both of the ocular adnexa (p = 0.03) and of the eyeball (p = 0.04). Patients over the age of 50 exhibited a six times greater chances of intense adnexal involvement (p = 0.002).

## Discussion

The prevalence of eye involvement secondary to HD varies greatly in the literature, from 6 to 100%, depending on the study population, the methods applied and the clinical presentations [5–7, 20, 24–26]. This study conducted refractional and clinical ophthalmological assessments and found at least one ocular abnormality in 86 (100%) patients. Although not all data were statistically significant, ocular involvement tended to be more accentuated in patients with virchowian and borderline forms and in those with some type of DFI. The findings follow the trend reported in other studies [7, 24–25], however we draw attention to the high prevalence of Meibomian gland dysfunction.

Even when HD cases are identified and MDT is implemented, there is evidence that about 20% of patients with MB have ocular pathologies with potential loss of vision at diagnosis, during treatment or within 5 years after completion of the treatment [6, 17]. Several studies have shown that patients continue to develop new eye complications after successful treatment completion and are believed to be related to ongoing immune reactions and the slow evolution of pre-existing nerve damage [6, 27]. Daniel et al found that, each year, approximately 5.6% of MB patients who completed treatment with MDT can develop new eye complications from leprosy, which are often (3.9%) potentially threatening to vision [27]. Although the cross-sectional design of our study does not make it possible to compare with the findings of these authors, we can observe that, in fact, the patients who have already completed the treatment, therefore considered microbiologically cured, did not have a statistically significant lower rate of ocular involvement compared with the patients who still are in MDT, therefore with active disease.

**Fig 3:**
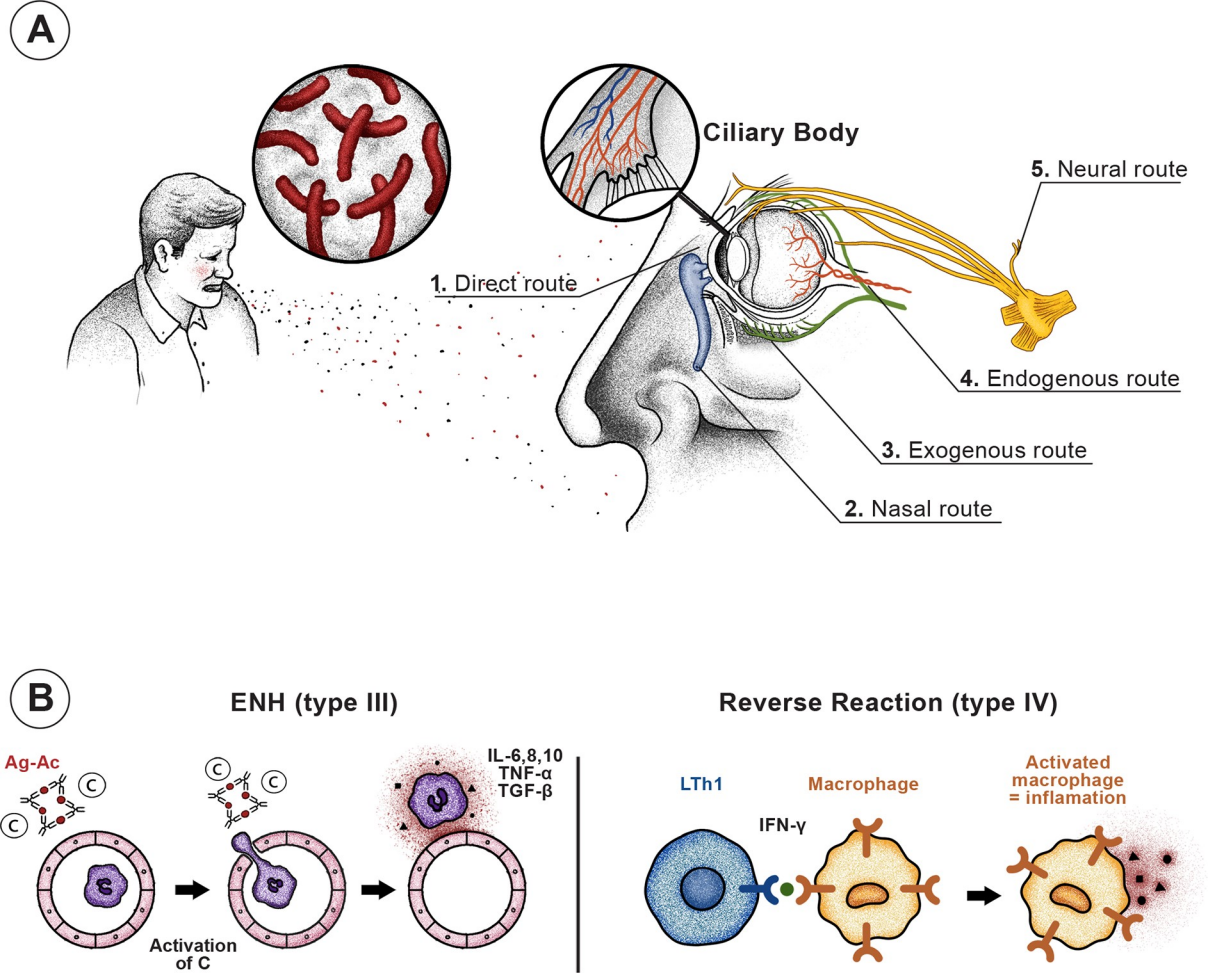
Mechanisms of ocular injury in Hansen’s disease. **(A) Primary injury to the ocular tissues by the Hansen’s bacillus, which can reach the eye: directly–inoculation by droplets eliminated from the airways of patients; nasally–the mycobacteria ascends via the nasolacrimal canal; neurally–invasion of ocular innervation via the trigeminal and/or facial nerves; endogenously–via the blood vessels of the ciliary body, where the bacillus multiplies and invades the iris, cornea, conjunctiva, etc.; exogenously–the bacillus arrives at the eye via perivascular and lymphatic channels in the skin and infiltrated subcutaneous cells. (B) Secondary injury of ocular structures caused by immunological reactions during reactional states–by deposition of immunocomplexes (Gell and Coombs type III) or by late hypersensitivity reaction (Gell and Coombs type IV).** ENL, erythema nodosum leprosum; Ag-Ac, antigen-antibody complex; C, complement; PMN, polymorphonuclear; IL, interleukin; TNF-α, tumor necrosis factor alpha; TGF-β, transforming growth factor beta; LTh1, Type 1 T helper lymphocytes; IFN-γ, interferon gamma.

The ocular signs and symptoms of HD can be caused by primary bacterial infection of ocular tissues by the bacillus–whether by direct inoculation or by hematogenic, neural (facial and trigeminal nerves), or lymphatic dissemination–and also by immune reactions including erythema nodosum leprosum (ENL) (Fig 3) [7, 8, 16, 24]. Although a significant number of patients (43%) exhibited some type of leprosy reaction during the course of the disease, it could not be determined whether the patient was in a reactional state at the time of the ophthalmological examination, since the examiner was blind to the patients’ clinical characteristics.

The visual acuity measurement is the most important item on the list of ocular findings that make up the WHO Ocular Disability Grading. In this study, just one patient exhibited unilateral vision loss and there were no cases of bilateral blindness caused by HD (vision worse than 0.1 or 20/200 in the eye with worse acuity). Other studies have reported percentages of blindness varying from 2 to 33% of patients with the disease [5–7, 20, 24–26]. The most common causes of blindness related to HD are corneal diseases secondary to lagophthalmos, chronic iritis, and cataracts [6, 7, 24, 25].

Lagophthalmos is caused by damage to the zygomatic branch of the facial nerve, which leads to weakness or paralysis of the orbicularis muscle of the eye [24]. It is classed as a G2I and was present in just one patient, in contrast with other studies, in which its occurrence ranged from 4 to 30% [7, 25]. Ocular involvement is directly related to disease duration. In studies that observed more severe ocular involvement, mean disease duration was as long as 15 years [25]. In our study, mean time since diagnosis was 3.3 years, which may explain the lower rate of severe injuries.

Thousands of people all over the world have problems with dry eye and this discomfort is one of the most frequent causes of patients’ visits to ophthalmological consultations. The “Dry Eye World Study” (DEWS II) defines the disease of the surface of the eye as multifactorial and characterized by instability of the tear film, tear hyperosmolarity, inflammation and damage of the surface of the eye, and neurosensory abnormalities of the cornea [28], the last of which is the principal factor among patients with HD [7]. This is compounded by injuries to the fibers of the intermediate nerve that follows the facial nerve and is responsible for the parasympathetic innervation of the lacrimal gland, causing reduced tear production, and potentiating the signs and symptoms of tear film abnormality [16].

Since there is no specific diagnostic test for dry eye disease, it is difficult to ascertain its prevalence. The first study of its prevalence and risk factors in a Brazilian population was published in 2018, using a simplified questionnaire on the symptoms of dry eye and reporting a prevalence of 12.8% [29]. The occurrence of diagnosis of dry eye among patients with HD in our study was 81.4%, of which 50% were due to evaporative causes, 19% due to low production, and 31% due to mixed causes. Another Brazilian study reported a prevalence of dry eye among patients with HD of 21.4% of cases, compared to 13.3% of controls, but without statistical significance (p = 0.429) [30].

The Meibomian glands are located in the posterior lamella of the eyelid border and play an important role in the physiology of the tear film. The oily secretion that they produce forms a film over the aqueous tear layer, reducing evaporation. Meibomian gland dysfunction is a term that refers to any type of functional abnormality and it has a high prevalence rate in the general population [31]. Ophthalmological examinations of people with this condition often reveal mechanical obstruction of the ostia of the glands by scaling of the epidermis at the eyelashes, telangiectasias of the eyelid border and, in chronic cases, trichiasis or distichiasis. The engorged glands initiate inflammatory activity, which can be compounded by bacterial infection. In HD, the final result of secondary infiltration and infection of these glands may be atrophy, compromising the quality of the tear film. Tear osmolarity is increased and mechanisms that defend the anterior surface of the cornea are lost [16, 30].

Meibomian gland dysfunction is the most common cause of evaporative presentations of dry eye, reducing the time taken for the tear film to be ruptured and the appearance of punctiform corneal lesions, primarily involving the lower third [32]. Meibomitis and blepharitis are common among patients with prolonged HD [16]. Meibomian gland dysfunction was the most frequent grade 1 ocular disability in the present study (89.5%). In these cases, visual acuity can also be affected, and the main cause is tear film instability [32].

Corneal sensitivity is an important stimulus of tear production. Corneal hypoesthesia compromises maintenance of the surface of the eye and of secretion by the primary and accessory lacrimal glands. Corneal hypoesthesia or anesthesia are primarily observed in long duration multibacillary forms and are caused by thickening of the corneal nerves in response to direct invasion by the bacillus [7]. All of the 11.6% of the patient sample who had an abnormal monofilament test result in our study had virchowian or borderline forms of the disease (p = 0.08). Among the eye surface changes observed, pterygium was found in 25.6% of the patients. Other studies have reported prevalence ranging from 4.6 to 21.7% [7, 33].

Iris atrophy and small pupils are ocular characteristics of HD [34, 35]. Atrophy of the iris is present in up to 25% of patients with MB HD and can be caused by invasion of the nerves by *M*. *leprae*, by inflammation due to leprosy reactions, and by aging. Patients over the age of 60 are 5 times more likely to develop atrophy of the iris than patients less than 20 years old [7].

Pupillary dysfunction is common in patients with HD, who characteristically present with persistent miosis, slow pupillary reaction to light, and poor pupillary dilation in response to anticholinergic mydriatic agents [36]. Pupil cycle time is an objective tool for assessment of the pupillary reflex arc and can be used to evaluate the integrity of afferent and efferent pathways and of the musculature of the iris. Studies have reported PCT for healthy controls at 0.820 s ± 47ms and 0.801ms ± 89ms, and report difference in PCT between the eyes of subjects as 30ms ± 13 and 30ms ± 36 [37]. Normal individuals aged from 12 to 50 years have PCT values lower than 0.900s and just 5% have values exceeding 0.954s or a difference between eyes exceeding 70ms [38]. Postganglionic autonomic neuropathy in patients with HD was demonstrated by increased PCT even in eyes assessed as normal [39]. In our study, 52.3% of the sample had PCT > 0.900s.

Cataract rates ranging from seven to 33% of patients with HD have been reported [7]. Although the exact mechanism is not known, presence of HD triples the risk of developing a cataract and occurrence of ENL increases this risk by around 6 times [15]. In our study, 22 (25.6%) patients exhibited cataracts, and the possibility that this is due to frequent use of corticoid therapy to treat leprosy reactions cannot be ruled out.

A low IOP is a common finding in patients with HD and is caused by destruction and late atrophy of the ciliary body due to *M*. *leprae* invasion, with consequent low production of aqueous fluid [16]. A case control study found a mean IOP of 14.2 mmHg in healthy controls and 13.1 mmHg in patients with HD (p = 0.01) [40]. Reduced IOP is also seen in the majority of patients with lepromatous iridocyclitis, as observed in another study, in which mean IOP was 10.1 mmHg and 11 mmHg in HD patients with and without chronic iridocyclitis, respectively [41]. In our study, mean IOP was 14 mmHg and 15% had a measurement less than or equal to 10 mmHg in at least one of the eyes.

In contrast with the finding of low IOP, glaucoma is an uncommon complication in HD and has been reported in 10% of patients with HD [42], which is similar to the finding in our study, in which 9.3% had this condition. In the majority of cases, glaucoma is secondary to immune reactions, with deposition of amyloid material in the ciliary body, sclera, and trabecular meshwork, compromising drainage of the aqueous humor. Another factor in the elevation of IOP in these patients is the frequent and prolonged use of corticosteroids, leading to development of cortisone glaucoma [16].

Involvement of the posterior segment of the eye or the optic nerve is extremely rare in HD. However, some reports suggest that there may be subclinical compromise of the optic nerve, particularly during leprosy reactions [43]. In order to better evaluate these structures, the patients in this study underwent OCT, but no abnormalities related to HD were detected. There are reports of use of this imaging exam modality to assess the anterior segment of the eyes of patients with HD, particularly uveal involvement, but there are no reports in the literature of using OCT for evaluation of the posterior segment in this group of patients in particular [44].

Among the limitations of this study is the lack of a control group to compare the ocular findings with subjects of the same age and sex. Even though we examined more than 80 patients, the findings cannot be generalized due to the convenience sampling used. A larger sample size potentially would be able to detect other possible complications of Hansen’s disease as more severe cases of visual acuity loss. Also, the presence of patients with DFI associated with ocular findings could have increased its association with our ophthalmological outcomes. However, it is essential to emphasize that only five patients in our sample had some degree of incapacity associated with ocular manifestations, according to the medical records where they are followed up. The strength is the complete ophthalmological examination of these patients adding new data about the manifestations of this still frequent granulomatous disease.

## Conclusions

The prevalence of ocular abnormalities was 100% in the patients with HD evaluated in this study. The most prevalent findings were Meibomian gland dysfunction and dry eye syndrome.

Of the variables analyzed, age greater than 50 years and presence of a DFI were statistically relevant factors with relation to presence of ocular abnormalities. Patients classified as having borderline or virchowian forms tended to exhibit more abnormalities than tuberculoid. Individuals with some DFI (1 or 2) had around a three times greater risk of intense abnormalities of both the adnexa and the eyeball. Patients still in treatment did not have greater ocular involvement than those who had already completed MDT and been taken off it because of cure.

Considering the elevated prevalence of ocular involvement among patients with HD, there is a need for greater interdisciplinary cooperation between dermatologists and ophthalmologists, ensuring these patients have greater access to periodic ophthalmological examinations.

## Supporting information

S1 Checklist STROBE checklist. (DOC)
